# Working memory and inattentive behaviour in a community sample of children

**DOI:** 10.1186/1744-9081-3-12

**Published:** 2007-02-23

**Authors:** Mariko Lui, Rosemary Tannock

**Affiliations:** 1 Human Development and Applied Psychology, Ontario Institute for Studies in Education of the University of Toronto, 252 Bloor Street West, Toronto, Ontario M5S 1V6, Canada; 2 Program in Neurosciences and Mental Health, The Hospital for Sick Children, 555 University Avenue, Toronto, Ontario M5G 1X8, Canada

## Abstract

**Background:**

Existing literature to date suggests a relationship between cognitive attention and working memory (WM), but the relationship between overt inattentive behaviour and WM is less clear. This study examined the relationship between WM and parent-rated inattentive behaviour in a community sample of 140 children aged 7–12 years.

**Methods:**

Children completed 2 clinical (laboratory-based) measures of WM (auditory-verbal and visual-spatial) and a measure of real-life WM, designed specifically for this study, while their parents completed questionnaires about their child's inattentive behaviour and other areas of functioning.

**Results:**

Findings indicated that poorer performance on WM tasks predicted inattentive behaviour.

**Conclusion:**

These results are consistent with previous research linking WM deficits and poor attention in ADHD and normal populations. The present findings support a controlled attention model of WM.

## Background

Working memory (WM) is a cognitive processing resource of limited capacity that allows the temporary storage of information while simultaneously processing the same or other information. Performance on WM measures correlates with performance on higher-order cognitive tasks involving reading comprehension, reasoning, and complex learning [1]. A well-known model of WM is Baddeley and Hitch's model [2,3], in which WM is comprised of two separate systems for the temporary storage of verbal information ('phonological loop') and visual-spatial information ('visuo-spatial sketchpad') and a 'central executive' as a control mechanism that manipulates the information in active storage in order to perform complex cognitive tasks. Many other theoretical perspectives on WM exist [4], but one area of general agreement is the distinction between processes involving storage (e.g., short-term memory; STM) and those involving greater cognitive control (e.g., central executive).

The controlled attention view of WM, proposed by Engle and his colleagues [5], is another model of WM that consists of STM and executive-attention components (consistent with Baddeley and Hitch's storage and central executive). However, instead of emphasizing how large the STM store or WM capacity (i.e., how much WM can hold), the controlled attention perspective views information maintenance *in the presence of interference *as a critical control function of WM, and hence as the primary mechanism linking WM capacity with higher-order cognition [5]. In other words, controlled attention is an executive control capability that can "effectively maintain stimulus, goal, or context information in an active, easily accessible state in the face of interference, to effectively inhibit goal-irrelevant stimuli or responses, or both" [6] [p. 180].

Studies of individual differences in WM provide support for the conceptualization of WM as controlled attention. For example, studies that compared high- and low-WM span individuals in interference resistance and processing load found that individuals with high WM ability are better able to resist interference during encoding and retrieval than individuals with poor WM ability [7,8]. Moreover, a recent investigation of the "cocktail party phenomenon" (which refers to a situation in which one can attend to only part of a noisy environment, yet highly pertinent stimuli such as one's own name can suddenly capture attention) indicated that individual differences in WM capacity is related to the ability to block out distracting information [9].

Collectively, previous research supports a relationship between controlled *cognitive *attention and WM, but is unclear whether WM would relate to overt inattentive behaviour (e.g., as manifested by individuals with Attention-Deficit/Hyperactivity Disorder [ADHD]). Examples of overt inattentive behaviour are: difficulty sustaining attention in tasks or play activities, being easily distracted by irrelevant stimuli, and frequently interrupting ongoing tasks to attend to trivial noises or events that are usually ignored by others [10]. Such a relationship would be important to study since the correspondence between overt inattentive behaviour and covert cognitive attention is unclear [11]. For example, one study showed that children's inattentive behaviour (i.e., looking away from the task) observed during a cognitive attention test did not correspond to any decrement in task performance[12]. However, other studies of children with ADHD report a relationship between inattentive behaviour and visual-spatial WM, but not with auditory-verbal WM [13-15].

The primary purpose of the present study was to examine the relationship between WM performance and inattentive behaviour in a community sample of school-aged children. According to the controlled attention model, poor/overloaded WM should lead to more interference, and hence, inattention/distraction. In line with this, we examined the correspondence between caregiver reports of inattentive behaviour and performance on two types of WM measures: clinical, laboratory-based measures of WM (auditory-verbal and visual-spatial) and a real-life measure of WM. The real-life measure of WM was included in this study to obtain a broad assessment of different aspects of WM. Because most studies of WM take place in a highly controlled laboratory setting in which interference from external sources is reduced and rigorous constraints are placed on the child's behaviour, performance may either exaggerate, or conversely, mask the true magnitude of a child's WM capacity [16]. In addition, many clinical measures of WM do not reflect critical everyday cognitive tasks, and hence cannot demonstrate ecological validity (the degree to which results obtained in a controlled experimental condition are related to those obtained in a real-life, naturalistic environment) [17]. Here, we seek evidence of a relationship between both types of WM (clinical and real-life) and children's inattentive behaviour.

The hypotheses for the present study were derived from the controlled attention view which argues that WM capacity is a valid predictor of (covert) attentional control, and that high- and low-span individuals allocate attention differently, with high-WM span individuals being more adept at resisting interference than their low-span counterparts [6]. The present study sought to extend this controlled attention perspective, by testing the general hypothesis that WM performance would be inversely related to caregiver ratings of overt behavioural inattention, such that children with better WM performance would be able to resist distraction on tasks and hence have lower ratings of behavioural inattention than children with poor WM performance.

A secondary goal was to test the more specific hypothesis that visual-spatial WM, but not auditory-verbal WM, would be inversely related to inattentive behaviour ratings, such that better performance on visual-spatial WM tasks would be associated with lower ratings of inattentive behaviour. This hypothesis was based on Chhabildas et. al's findings [13] that ADHD inattentive behaviour is related to visual-spatial WM. As corollary hypotheses, we predicted that WM would not be associated with parent ratings of hyperactivity/impulsivity or any other emotional or behavioural difficulties.

## Methods

### Participants

A total of 140 children aged 7 to 12 years participated in this study. The mean age of the sample was 10.03 years (SD = 1.72), and approximately half of the sample were females (n = 72). Information from the demographic questionnaire completed by caregivers indicated that approximately 62% of the participants were Caucasian, and the remaining participants were relatively evenly distributed across African-Americans (6%), Asians (6%), South Asian (3%), and other (20%), consistent with the ethnic representation of the large metropolitan city. Of the total sample, caregivers reported 98% of the children's primary language was English, 88% reported no learning or developmental disability, and 93% reported no significant hearing or vision problems. All participants were included in the study, as there were no inclusion/exclusion criteria, other than age, English spoken as primary language at home, and independence in mobility (i.e., able to get around the water park without the need for physical assistance).

The sample was recruited during a two-week period in the summer from visitors to a large water park just outside an urban city. A water park was chosen as the site of research data collection because it attracted many children in the appropriate age-range for the present study. As a thank-you gift, all participants received a pencil and a voucher for free items available from food stations on the park grounds.

### Measures

The measures for this study included clinical measures of auditory-verbal and visual-spatial WM (both having a 'storage' component and a 'central executive' component), and a real-life measure of WM for the children, and questionnaires for the parents.

#### Clinical measures of working memory for children

The clinical measures of WM consisted of the Digit Span subtest of the Wechsler Intelligence Scale for Children, Third Edition (WISC-III) [18] and the Spatial Span subtest of the WISC-III – Process Instrument (WISC-III PI) [19].

The Digit Span subtest is a clinical measure of auditory-verbal WM. Children heard a sequence of digits (recorded onto audio tape; participants listened through headphones to reduce interference from distracting sounds from the water park) at a rate of one digit per second, and were asked to repeat the sequence of digits in the exact order it was presented. The length of the sequence started with two digits, and became increasingly more difficult (up to a maximum of nine digits) until the children obtained the required number of errors for discontinuation. The raw score for Digit Span Forward (DSF) is the number of correct trials (maximum raw score of 16 correct trials). Digit Span Forward is a measure of verbal storage-only component of WM (phonological loop component of Baddeley's multicomponent model of WM; see ref [20] for construct validity of DSF).

For Digit Span Backward (DSB), children heard a sequence of digits (again, through headphones) that they were required repeat in reverse order. The sequences became increasingly more difficult (from a sequence of two digits to a maximum of nine digits) until the children obtained the required number of errors for discontinuation. The raw score of DSB is the number of correct trials (maximum raw score of 14 correct trials). Digit Span Backward is a measure of the storage and processing components of verbal WM (i.e., phonological loop + central executive components of WM; see [20] for construct validity of DSB).

The Spatial Span subtest is the visual-spatial version of Digit Span. It uses a Spatial Span board, upon which 10 blue cubes are mounted randomly. For Spatial Span Forward (SSF), the researcher tapped the cubes (one cube per second) in a specified sequence that the children were asked to replicate. The length of the sequence started with two blocks, and became increasingly more difficult (to a maximum sequence of eight blocks) until they obtained the required number of errors for discontinuation. The raw score for SSF is the number of correct trials (maximum score of 14 correct trials). Spatial Span Forward is a measure of the visual-spatial storage component of WM (i.e., visuo-spatial sketchpad) [21].

For Spatial Span Backward (SSB), the researcher tapped the cubes in a specified sequence that the children were required to repeat in reverse order. Like SSF, the length of the sequence became increasingly more difficult (from a sequence of 2 blocks to a maximum sequence of 8 blocks) until they obtained the required number of errors for discontinuation. The raw score for SSB is the number of correct trials (maximum score of 14 correct trials). Spatial Span Backward is a measure of the storage and processing components of visual-spatial WM (i.e., visuo-spatial sketchpad + central executive components of WM) [21].

In all statistical analyses that follow, only raw scores were used for DSF, DSB, SSF, and SSB. Scaled scores are not reported here.

#### Real-life measure of working memory for children

The Key Search task is an experimental measure of real-life WM, designed for the purpose of the present study. This task was the real-life version of Digit Span and Spatial Span, and was individually administered. For Key Search Forward (KSF), children were instructed to visit a certain number of places in the water park (e.g., locker area, wave pool, waterslide). Verbatim instructions given to each participant are presented in Appendix A. While giving verbal instructions as to which places to visit on a given trial, the researcher simultaneously pointed to each place on a laminated map of the water park. This step, along with an initial screening of children's familiarity with the layout of the park (described below), was included to address the possible impact of way-finding ability. Children were instructed to visit each place (with a researcher) in the given order. For example, in trial 1 of KSF, the participant was instructed to go to the hot tub, the lazy river, and the lockers. Note that the places were not all visible from the start point and the Park map was not available to the child after the initial instructions. Thus, participants had to maintain and update that information as they walked from one place to the other. A score of 1 was assigned if the child visited all the places in correct order within a trial (else, a score of 0). The raw score for KSF is the number of correct forward trials (maximum raw score of 5 correct trials). Because the children may have either engaged in verbal rehearsal of the places and/or constructed an internal visual-spatial map, KSF was thought to be a real-life measure of both auditory-verbal and visual-spatial STM, or storage component of WM.

When the children completed a KSF trial (i.e., at the last destination of a given forward trial that the child could recall), the accompanying researcher asked them to imagine that somewhere on the way to the final destination, the researcher lost his/her locker key. For Key Search Backward (KSB), the researcher asked the children to help find the key by re-tracing their steps. Appendix A displays the exact instructions given to each participant. The children verbally reported all the places they visited in backward order. A score of 1 was assigned if they correctly recalled all the places they visited in reverse order (else, a score of 0). The raw score for KSB is the total number of correct backward trials (maximum raw score of 5 correct trials). Key Search Backward is thought to be a naturalistic measure of both auditory-verbal and visual-spatial WM. All Key Search scores described in the Results section below are raw scores.

Unlike Digit Span and Spatial Span which both require the administration of all trials of the forward task until the discontinuation criteria had been met, after which all trials of the backward task are administered (i.e., first DSF, then DSB; first SSF, then SSB), the Key Search task required the forward and backward trials to be administered in alternating order. That is, after trial 1 of KSF was completed, trial 1 of KSB was administered. If a child received a score of 1 on either of the first trials of KSF or KSB, the child proceeded onto the second trials of KSF and KSB. Children started with a sequence of three places to visit (trial 1). The sequences became increasingly more difficult (4 places, 5 places, and so on) until they received a score of zero on both KSF and KSB in a given trial, at which time the Key Search task was discontinued. Finally, in order to discount the confound of familiarity with the layout of the water park, researchers screened children's knowledge of the water park before commencing the Key Search task. To do this, researchers asked the children to point in the direction of prominent landmarks (or indicate their proximity to visible landmarks) within the water park. All landmarks in the screening were included as places to visit in the actual Key Search task. If they pointed incorrectly, the researcher scored the item as zero, and corrected them by pointing in the correct direction of the landmark. A total score of zero on the screen indicates poor knowledge of the water park layout, while a perfect score of 6 indicates very good knowledge of the water park layout. Performance on the Key Search Screen was entered as a covariate in the relevant analyses to control statistically for way-finding abilities.

The real-life Key Search task and two clinical tasks differed in several important ways. One important difference lies in the sequence of administration of the forward and backward tasks as discussed in the Methods section. Another difference between the clinical WM tasks and the real-life WM task is the time it took to administer. Specifically, while the duration for the Digit Span and Spatial Span tasks is typically under a minute for each trial, the duration for the Key Search task was approximately a few minutes for each trial (participants heard which places to visit at about one place per second, then approximately two minutes to walk to all locations, and finally to recite the places in reverse order). Given this difference in administration time, it could be argued that episodic or long-term memory was used in the Key Search task. However, the Key Search task was developed to be analogous to everyday tasks that tap into WM processes, and not identical to laboratory-based WM tasks. Real-life tasks that tap into WM typically have longer durations than Digit Span or Spatial Span. For example, a teacher may instruct a student to first copy task instructions from the board, take out the necessary workbooks and work materials, and then to start the necessary task. Another example would be reading comprehension, where the student would be required to read the text, understand the material, continually update information that he/she just read, and then to answer content or inferential questions based on the text. Thus the Key Search Task approximates the distracting conditions and duration under which children are typically required to engage working memory abilities in everyday activities. In short, the Key Search task was developed to be analogous to the clinical WM tasks (storage and manipulation of information), yet also to be similar to WM tasks that children may encounter in their daily lives. More research assessing the ecological validity of neuropsychological measures (such as WM tasks) is needed to evaluate the generalizability of such tasks.

#### Questionnaires for parents

Parents completed three questionnaires about their children in three areas of interest: demographic information, a measure of inattention and hyperactivity/impulsivity, and a measure of psychological adjustment.

The demographic questionnaire contained questions about the parent and child's background, such as parent education, child health (e.g., vision or hearing problems, any medications, etc.), known learning disabilities or developmental disabilities, primary language, and ethnicity.

The Strengths and Weakness of ADHD-symptoms and Normal-behaviour (SWAN) scale was used to assess inattention and hyperactivity/impulsivity [22]. This scale has nine inattention items and nine hyperactive/impulsive items. Parents rated each item on a 7-point scale (Far Below Average = 3, Below Average = 2, Slightly Below Average = 1, Average = 0, Slightly Above Average = -1, Above Average = -2, and Far Above Average = -3). The nine inattentive scores were summed and then averaged for each participant, giving the Inattention score, and the nine hyperactive/impulsive scores were summed and averaged, giving the Hyperactivity/Impulsivity score. Thus, the higher the score, the more inattentive or hyperactive/impulsive the caregiver rated the child. The SWAN scale was chosen for this study because it captures the population variation presumed to exist in nature but truncated by the wording and scoring of items of other scales of difficulty (e.g., Not at All = 0, Just a Little = 1, Pretty Much = 2, Very Much = 3). The SWAN scale assesses strengths as well as weaknesses in individual cases, thereby producing a better degree of variability of inattention and hyperactivity/impulsivity in the population. Hence, for the purpose of this study, inattentive behaviour and hyperactive/impulsive behaviour are viewed on a continuum, rather than as clinical cut-off scores.

To ascertain the psychological adjustment of the participants, as well as to explore the extent to which WM is associated with other types of difficulties other than hyperactivity-inattention (Hypothesis 1), parents were asked to complete a short questionnaire to screen for emotional and behavioural difficulties. The Strengths and Difficulties Questionnaire (SDQ) [23] is a brief, one-page screening measure for parents of children aged 3–16 years. The SDQ asked about 25 attributes, some positive and others negative; parents used a 3-point Likert scale to indicate how far each attribute applied to their child. The 25 items were divided between five scales of five items each, generating scores for emotional symptoms, conduct problems, hyperactivity/inattention, peer relationship problems, and prosocial behaviour. Scores for each scale range from 0 (no problems in a given area) to 10 (indicates clinical range of functioning for a given scale). Studies have cited its usefulness as a screening instrument for psychiatric disorders [24]; in addition, studies have shown the SDQ to have adequate reliability, and discriminant and predictive validity [25,26].

### Procedure

A booth was set up in a highly visible area of the water park and signage was posted throughout the park. The signs contained a fun and interest-provoking logo inviting park guests to participate in the study. All research staff involved with this study wore special T-shirts for identification purposes. Research staff attended the booth at all times to provide information about the study and to complete consent procedures. Parents provided written informed consent for their child to participate and children provided informed verbal assent. The study was approved by Ethical Review Boards at the University and Hospital Research Institute as well as by the Water Park. All test activities were conducted on dry land outside of the water areas. Children were tested individually and were accompanied by a researcher throughout the whole test session.

Clinical WM testing took place in a partitioned part of the water park, in a tented area to simulate a quiet laboratory testing space. There were two partitioned "laboratories" with a table and two chairs in each. To reduce possible interference from noise from the water park during the verbal memory task, children listened to a pre-recorded audiotape of the verbal stimuli (i.e., digits) which were presented through headphones. Thus, testing procedures for the clinical WM tasks were slightly modified to accommodate the naturalistic setting of the study. While the children completed the clinical and real-life WM tasks, parents were asked to complete some questionnaires about their child. The Digit Span, Spatial Span, and Key Search tasks were administered in counter-balanced order.

### Statistical analysis

In the first step, we screened the sample for ethnicity, parent-reported health problems (e.g., learning disabilities, head injury), and psychological adjustment in the children and then investigated whether these problems influenced the findings. A total of 27 children were reported by caregivers to have health problems. To examine whether these self-reported problems had any effects on the results, all analyses were run with and without the 27 children. Virtually all results remained unchanged (i.e., no changes in significant findings); therefore, in the absence of clear evidence that these 27 participants were not part of a normal population, the following analyses included all 140 participants. In addition to screening for the difficulties stated above, the present study also screened for psychological adjustment in this community sample of children. The SDQ (North American version) yielded scores for five areas of emotional and behavioural difficulties. The proportion of caregivers reporting emotional and behavioural problems falling within the clinical range of each SDQ scale were as follows: 11% emotional symptoms; 9% conduct problems; 8% hyperactivity/inattention; 13% peer relationship problems; and 4% prosocial behaviour. The SDQ parent ratings for each scale approximated the proportion of children in the clinical range according to the SDQ norms (Normative SDQ data from Britain – % in clinical range: 11.3% emotional symptoms, 12.7% conduct problems, 14.6% hyperactivity/inattention, 11.8% peer problems, and 2.4% prosocial behaviour).

The second step was to examine the factor structure of the SWAN ADHD scale to determine the robustness of the inattention and hyperactivity/impulsivity scales, since this is a relatively new instrument and one that has not been investigated in a Canadian sample of children. A factor analysis, using a varimax rotation, was performed on the SWAN scale to check whether the data would yield the two proposed dimensions (i.e., inattention and hyperactivity/impulsivity). The results confirmed two separate factors with Eigen values greater than 1: the Eigen value for the hyperactive/impulsive factor was 9.36, which accounted for 52.0% of the variance, and the Eigen value for the inattentive factor was 2.40, which accounted for 13.3% of the variance. Results confirmed that all nine inattentive items have significant loadings with the inattentive factor, while none of the nine hyperactive/impulsive items have significant loadings. Likewise, all nine hyperactive/impulsive items have significant loadings with the hyperactive/impulsive factor, while none of the inattentive items have significant loadings (see Additional File 1 for the factor loadings for each item on the SWAN scale). Thus, entering the two constructs as separate variables in the planned correlational and regression analyses is validated.

As a third step, we conducted zero-order correlational analyses and multivariate analyses of variance (MANOVA) to investigate age and gender differences in the caregiver behavioural ratings. The fourth step addressed our primary objectives. We first conducted correlational analysis and a factor analysis to determine whether the six measures of WM we had selected did in fact index the purportedly separable components of WM and then used regression techniques to test the hypotheses concerning the relationship between WM and inattentive behaviour. In view of the multiple comparisons to be made, alpha was set at .01 in all analyses to reduce the likelihood of Type I errors.

## Results

### Age and gender analyses

#### Age and gender analyses for WM measures

Zero-order correlations revealed that age correlated significantly with all WM raw scores (for DSF, r = .53, p < .001; for DSB, r = .55, p < .001; for SSF, r = .55, p < .001; for SSB, r = .50, p < .001; for KSF, r = .52, p < .001; and for KSB, r = .54, p < .001) such that WM performance on all tasks improved significantly with age (as expected with the use of raw scores instead of scaled scores). To examine the effects of gender (male, female), a one-way multivariate analysis of variance (MANOVA) was conducted on all WM measures (DSF, DSB, SSF, SSB, KSF, and KSB raw scores). Table 1 displays the means and standard deviations for males and females on all dependent measures. No significant differences were found among males and females on the WM measures, Wilks's Lambda (Λ) = .92, F(6, 132) = 2.02, p = .07. Follow-up analyses of variance (ANOVAs) on each dependent variable revealed no significant differences between boys and girls on WM performance.

**Table 1:** Means and standard deviations on WM measures, SDQ scales, and SWAN scales for males and females

	*Males*		*Females*	
	*Mean*	*SD*	*Mean*	*SD*
Digit span forward	8.40	1.96	7.83	2.10
Digit span backward	4.87	1.82	5.00	1.69
Spatial span forward	6.89	2.05	6.58	2.02
Spatial span backward	5.87	2.19	5.92	2.17
Key search forward	1.42	.89	1.50	.96
Key search backward	1.27	.85	1.46	.95
Key search screening	5.39	.92	5.21	.98
SDQ Emotional symptoms	1.81	1.91	1.75	1.90
SDQ Conduct problems	1.34	1.63	1.31	1.36
SDQ Hyperactivity – Inattention*	3.41	2.44	2.42	2.06
SDQ Peer relationship problems	1.69	1.97	1.43	1.63
SDQ Prosocial behavior	8.11	1.80	8.56	1.81
SWAN Inattentive*	-.44	.93	-.92	.94
SWAN Hyperactive/Impulsive	-.51	1.11	-.86	.96

*Note*: SDQ = Strengths and Difficulties Questionnaire; SWAN = Strengths and Weakness of ADHD-symptoms and Normal-behavior. *Significant difference between males and females, p < .01

#### Age and gender analyses for SWAN

While age was not significantly correlated with SWAN Inattention (r = -.19, p = .03), age was significantly correlated with SWAN Hyperactivity/Impulsivity (r = -.25, p < .01) suggesting that as children's age increased, parent ratings of hyperactivity/impulsivity decreased significantly. A one-way MANOVA was conducted to determine the effect of gender (male, female) on the two dependent variables (SWAN Inattention average, SWAN Hyperactive/Impulsive average). Gender differences approached significance on the SWAN scales, Wilks's Λ = .94, F(2, 139) = 4.70, p = .011. Follow-up ANOVAs revealed a significant difference between males and females on SWAN Inattention only, F(1, 140) = 9.45, p < .01, such that parents rated boys as more behaviourally inattentive than girls (see Table 1).

#### Age and gender analyses for SDQ

As with the SWAN ratings, analyses were conducted to examine age and gender differences on SDQ ratings. Zero-order correlations revealed that children's age did not correlate significantly with any of the SDQ scales (for emotional symptoms, r = -.07, p = .38; for conduct problems, r = -.13, p = .13; for hyperactivity-inattention, r = -.16, p = .06; for peer relationship problems, r = .07, p = .43; and for prosocial behaviour, r = .04, p = .64). To examine the effects of gender (male, female), a one-way MANOVA was conducted on SDQ ratings (emotional symptoms, conduct problems, hyperactivity-inattention, peer problems, and prosocial behaviour). Although no significant differences were found among male and females on the SDQ scales in the multivariate analysis, Wilks's Λ = .93, F(5, 136) = 1.96, p = .09, we proceeded with follow-up ANOVAs to test for expected gender differences in hyperactivity/inattention and emotional symptoms. Univariate analyses revealed that the hyperactivity-inattention scale was the only SDQ scale that showed a significant difference between males and females, F(1, 140) = 6.94, p < .01, such that parents rated boys as more hyperactive-inattentive than girls (see Table 1).

### Working memory and inattention

A zero-order correlation analysis was performed to examine the relationships among the six WM measures (DSF, DSB, SSF, SSB, KSF, and KSB raw scores), as well as with SWAN Inattention and Hyperactivity/Impulsivity scores. As can be seen in Table 2, all WM tasks correlated significantly with each other, suggesting that children who perform better at one WM task will generally perform better at the others. Moreover, results of the factor analysis, which tested the separability of the WM measures, revealed only one component with an Eigen value greater than 1 (Eigen value = 3.51), with all measures loading highly on the one extracted factor (DSF = .68, DSB = .76, SSF = .77, SSB = .73, KSF = .81, KSB = .84). Accordingly, a composite WM score was computed (i.e., the average of all 6 WM scores) for each participant. This finding precluded our secondary aim to test the specific hypothesis of the relationship between visual-spatial WM and Inattentive behaviour: thus we proceeded with analyses to test our primary hypothesis regarding WM (composite) and Inattentive behaviour (reported below).

**Table 2:** Zero-order (Pearson) Intercorrelations Between SWAN Inattention, SWAN Hyperactivity/Impulsivity, Auditory-Verbal, Visual-Spatial, and Real-Life Working Memory

	1	2	3	4	5	6	7	8
1. SWAN Inattention Average	-----							
2. SWAN Hyperactivity/Impulsivity Average	.64*	-----						
3. Digit Span Forward raw score	-.18	-.24*	-----					
4. Digit Span Backward raw score	-.22*	-.26*	.49*	-----				
5. Spatial Span Forward raw score	-.09	-.08	.45*	.51*	-----			
6. Spatial Span Backward raw score	-.34*	-.25*	.49*	.50*	.57*	-----		
7. Key Search Forward raw score	-.20	-.17	.36*	.45*	.49*	.36*	-----	
8. Key Search Backward raw score	-.29*	-.18	.37*	.48*	.51*	.44*	.92*	-----

* p < .01.

To test the main hypothesis (that WM performance would predict behavioural inattention), the first multiple regression analysis had SWAN Inattention scores as the dependent variable, and examined the significance of the unique variance added to the equation by WM performance over and above that which could be accounted for by age and gender. Age and gender were entered as predictors in step 1 and WM composite score as a predictor in step 2. Table 3 displays the standardized regression coefficients (β), the t-scores for β, R square change (ΔR2), and F change (ΔF). As shown in Table 3, children's gender contributed significant unique variance to SWAN Inattention in step 1. The R square change was significant for Step 2, ΔF(1, 135) = 8.58, p < .01, indicating that the WM composite contributed a significant amount of unique variance to SWAN Inattention ratings over and above age and gender. This finding is consistent with the main hypothesis that WM performance would predict parent ratings of inattention.

**Table 3:** Multiple regression analysis predicting SWAN Inattention

	β	*t *for β	ΔR^2^	ΔF
Step 1 Predictors:				
Age	-.21	-2.56*	.11	8.24**
Gender	-.26	-3.19**		
				
Step 2 Predictor:				
WM composite	-.32	-2.93**	.05	8.58**

*p < .05; **p < .01

Next, we tested the corollary hypothesis that WM would not be associated with parent ratings of hyperactivity/impulsivity. Therefore, the second multiple regression analysis had SWAN Hyperactive/Impulsive ratings as the dependent variable to determine whether WM performance was a valid predictor over and above age and gender. As shown in Table 4, children's age was the only significant predictor of SWAN Hyperactivity/Impulsivity (in step 1), while the WM composite score was not a significant predictor in step 2.

**Table 4:** Multiple regression analysis predicting SWAN Hyperactivity/Impulsivity

	β	*t *for β	ΔR^2^	ΔF
Step 1 Predictors:				
Age	-.25	-3.10*	.09	6.83*
Gender	-.17	-2.07		
				
Step 2 Predictor:				
WM composite	-.19	-1.71	.02	2.94

*p < .01

To test the second corollary hypothesis that WM would be associated only with inattentive behaviour and not with other psychological difficulties, we conducted a zero-order correlation analysis between the composite WM score and the five SDQ scales (emotional symptoms, conduct problems, hyperactivity, peer relationship problems, and prosocial behaviour). It is important to note that the hyperactivity subscale includes 2 items for inattentive behaviour as well as three hyperactivity items. As predicted, there were no significant correlations between WM and SDQ Emotional Symptoms scale (r = -.16, p > .01), SDQ Conduct Problems scale (r = -.12, p > .01), SDQ Hyperactivity scale (r = -.15, p > .01), SDQ Peer Problems scale (r = -.01, p > .01), and SDQ Prosocial scale (r = .03, p > .01).

## Discussion

Our primary goal was to test the hypothesis that poor WM would lead to susceptibility to interference, and thus be associated with higher behavioural ratings of inattention. Assuming that both WM and inattentive behaviour are continuously distributed traits in the normal population, we investigated this relationship in a community sample of school-aged children. Consistent with this hypothesis, we found that the children's WM performance predicted significant and unique variance in behavioural inattention as rated by their caregivers, over and above the contribution of age and gender. The finding that WM performance did not contribute significantly to parent ratings of hyperactivity/impulsivity and other behavioural/emotional problems, adds additional support to the hypothesis. Our findings are consistent with the controlled attention view of WM, which argues that the critical issue is not how much information WM can hold, but rather it is the extent to which executive control protects the information being processed from interference by irrelevant distractors. Findings from the present study also extend the controlled attention model of working memory by testing the predictions using parent ratings of inattentive behaviour (instead of covert attentional processes). In the present study, children who exhibited weaker WM executive control were thought to have weaker ability resisting interference, which manifested as inattentive behaviour, as viewed by their caregivers.

This finding converges with previous demonstrations that increased WM load results in greater distractor interference in Stroop-like tasks [27,28], which also support the controlled attention hypothesis that WM provides goal-directed control of manipulation allowing for minimal interference by goal-irrelevant distractors.

Our primary hypothesis regarding the link between inattention and WM also required evidence that that WM performance was *not *associated with ratings of hyperactivity/impulsivity or with other emotional/behavioural difficulties. A follow-up multiple regression analysis revealed that the composite WM score did not significantly predict SWAN ratings of hyperactivity/impulsivity. Moreover, a zero-order correlational analysis revealed that the WM composite score did not correlate significantly with any of the five SDQ behavioural/emotional scales at the set alpha level.

Another aspect of this study was to evaluate the relationship between experimental measures of real-life WM (i.e., KSF and KSB) and behavioural inattention. Given that the factor analysis failed to extract a separate factor for our real-life WM measure, this relationship could not be explored statistically. However, this finding of one factor may be an indication of ecological validity of the clinical measures of WM. Further investigation (with more rigorous statistical analyses) into the ecological validity of the clinical measures of WM used in this study is recommended for future studies.

Other findings from this study concerned age and gender effects of behavioural ratings. One analysis revealed that as children's age increased, parent ratings of SWAN Hyperactivity/Impulsivity decreased. An examination of gender effects revealed that parents rated boys as more hyperactive-inattentive than girls on the SDQ and more behaviourally inattentive on the SWAN. These findings are consistent with epidemiological studies of ADHD indicating that the ratio of males to females in non-referred samples ranges from 2.5:1 to 5.1:1 [29].

Another limitation of this study is that our measure of behavioural inattention came from parent report only. Because children spend a large part of their day in school, teachers often provide valuable information regarding children's level of attention and behaviour. However, it was not feasible to obtain teacher reports of inattention for this study given that it took place in the summer when children do not attend school. Although studies have shown that parent-teacher concordance rate for ADHD is relatively poor [30], cross-informant agreement is not as poor for inattentive symptoms. For example, Crystal et al. [31] found that parent-teacher disagreement was significant for conduct problems, depression, and hyperactivity; there were no significant differences for attention problems. In addition Power et al. [32] found that combining teacher and parent ratings on the Inattention factor was not as useful as a single informant approach in ruling out diagnoses of ADHD.

Finally, the main finding from this study revealed that WM is associated with caregiver reports of inattentive behaviour. However, it should be noted that other cognitive causes of inattentive behaviour should also be investigated. For example, research has revealed that groups of ADHD children demonstrate high degrees of between-subject variation in performance almost irrespective of task or setting [33]. Such group heterogeneity has led to the suggestion of the existence of independent multiple pathways to ADHD [34]. One such possible causal pathway contributing to the heterogeneity of ADHD includes altered dopaminergic function as described by the dynamic developmental theory put forth by Sagvolden [35]. Thus, deficient WM may only be one factor contributing to inattentive behaviour.

### Theoretical and clinical implications

A major finding of this study was a link between WM and *behavioural *inattention in a community sample of children. Other studies have found that covert *cognitive *attention, or inattention, can predict individual differences in WM [7-9]. The present findings add to, and support, theoretical models of WM, such as the controlled attention view of WM by providing evidence of a link between WM and behavioural symptoms of inattention in a community sample of children. A controlled attention interpretation of our findings would suggest that children's over-loaded WM (or weak WM performance) enabled interference of goal-directed processing to occur, which manifests as behavioural inattention.

Our finding of a cognitive correlate of inattention in a community sample of children has important clinical implications. Mainly, problems with inattention in children are considered to be a developmental risk factor and are associated with poor achievement in reading and general cognitive delays [11,36]. Our results provide valuable information about the cognitive profiles of inattentive individuals, and point to possible educational remediation strategies. Warner-Rogers et al. [11] suggest that because children with pure inattentive behaviour are not likely to exhibit conduct problems, the educational and mental health needs of these children are at risk for being neglected. Future research is needed to underscore the importance of identification and treatment of children with inattentive difficulties.

## Conclusion

The present study provides empirical evidence of a significant link between WM performance and inattention in a community sample of school-aged children. Specifically, the results suggest that children manifesting poorer performance on WM tasks are rated by their parents as more inattentive behaviourally than are children with better WM. Directions for future research include obtaining teacher reports of children's behavioural inattention, as children's ability to sustain and focus attention is highly relevant within the classroom environment.

## Competing interests

The author(s) declare that they have no competing interests.

## Authors' contributions

This study was part of ML's master's thesis project. ML conceived of and designed the study, coordinated data collection, performed statistical analyses, and drafted the manuscript. RT is the thesis supervisor of ML, and made substantial contributions to the conception and design of the study, interpretation of the data, and revised the manuscript critically for important intellectual content. All authors read and approved the final manuscript.

## Appendix A

Key Search Instructions

Key Search Forward (Trial 1)

For this task, I'm going to tell you some places in the park, and show you where they are on this map. Listen carefully because I will say the places only once. When I'm done, I want you to *take *me to each place in the *same *order I tell you. For example, if I say "hot dog cart and ice cream stand," I want you to walk me first to the hot dog cart and then the ice cream stand. Do you understand? So, if I say "showers and hot tub," where would you take me first? Second?

Key Search Backward (Trial 1)

You did a great job taking me to the places I told you. But, let's imagine I lost my keys somewhere along the way and we would have to search for them in the all places we just went to. I want you to help me re-trace our steps by telling me all the places we went to in BACKWARDS order, starting from here, then the second last place, and so on.

Prompt: Now, where were we just before here?

Key Search Forward (Trials 2 & up)

OK, we're going to do the same thing: I'm going to tell you some places in the park, and show you where they are on this map. Listen carefully because I will say the places only once. When I'm done, I want you to take me to each place in the *same *order I tell you. Ready?

Key Search Backward (Trials 2 & up)

Once again, you did a great job taking me to the places I told you. As before, I want you to imagine that I lost my keys, and we must search for them in all the places we just went to. I want you to help me re-trace our steps by telling me all the places we went to in BACKWARDS order, starting from here, then the second last place, and so on.

Prompt: Now, where were we just before here?

Special Instructions

- Do not lead the child. Stay either beside or a little behind the child.

- Children are not to go in the water when doing this task. Tell the child to walk around the water.

## Supplementary Material

Additional File 1 Factor Loadings for Each SWAN Scale Item. The data provided represent the factor loadings for the Inattention scale and the Hyperactive/Impulsive scale on the SWAN Scale. Click here for file
