# Supplementary material for: Working memory and inattentive behaviour in a community sample of children
Source: Behav Brain Funct. 2007 Feb 23;3:12. doi: 10.1186/1744-9081-3-12 (PMC1820786; doi:10.1186/1744-9081-3-12)
Supplement: Additional File 1 — Factor Loadings for Each SWAN Scale Item. The data provided represent the factor loadings for the Inattention scale and the Hyperactive/Impulsive scale on the SWAN Scale. [file 1744-9081-3-12-S1.doc]

Additional File 1

Factor Loadings for Each SWAN Scale Item

| SWAN Item | Inattention  Factor | Hyperactive/ Impulsive Factor |
| --- | --- | --- |
| 1. Give close attention to detail and avoid careless mistakes | **.70** | .27 |
| 2. Sustain attention on tasks or play activities | **.73** | .34 |
| 3. Listen when spoken to directly | **.69** | .36 |
| 4. Follow through on instructions and finish school work or  chores | **.83** | .18 |
| 5. Organize tasks and activities | **.82** | .18 |
| 6. Engage in tasks that require sustained mental effort | **.74** | .13 |
| 7. Keep track of things necessary for activities | **.79** | .21 |
| 8. Ignore extraneous stimuli | **.63** | .39 |
| 9. Remember daily activities | **.73** | .28 |
| 10. Sit still | .20 | **.80** |
| 11. Stay seated | .38 | **.71** |
| 12. Modulate motor activity | .26 | **.71** |
| 13. Play quietly (keep noise level reasonable) | .17 | **.87** |
| 14. Settle down and rest (control constant activity) | .22 | **.82** |
| 15. Modulate verbal activity (control excess talking) | .23 | **.81** |
| 16. Reflect on questions (control blurting out answers) | .24 | **.81** |
| 17. Await turn (stand in line and take turns) | .33 | **.74** |
| 18. Enter into conversations and games without interrupting  or intruding | .31 | **.74** |
